# Long-term live cells observation of internalized fluorescent Fe@C nanoparticles in constant magnetic field

**DOI:** 10.1186/s12951-019-0463-5

**Published:** 2019-02-06

**Authors:** Anastasiia Garanina, Igor Kireev, Oxana Zhironkina, Olga Strelkova, Anton Shakhov, Irina Alieva, Valery Davydov, Sankaran Murugesan, Valery Khabashesku, Alexander Majouga, Viatcheslav Agafonov, Rustem Uzbekov

**Affiliations:** 1 GREMAN, UMR CNRS 7347, Université de Tours, 37200 Tours, France; 2 National University of Science and Technology «MISiS», 119049 Moscow, Russian Federation; 3 Faculty of Chemistry, Lomonosov Moscow State University, 119991 Moscow, Russian Federation; 4 Belozersky Institute of Physico-Chemical Biology, Lomonosov Moscow State University, 119234 Moscow, Russian Federation; 5 L. F. Vereshchagin Institute for High Pressure Physics of the RAS, 142190 Troitsk, Russian Federation; 6 Center for Technology Innovation, Baker Hughes a GE Company, Houston, TX 77040 USA; 7 D. Mendeleev University of Chemical Technology of Russia, Moscow, 125047 Russian Federation; 8 Faculté de Médecine, Université François Rabelais, 37032 Tours, France; 9 Faculty of Bioengineering and Bioinformatics, Lomonosov Moscow State University, 119192 Moscow, Russian Federation

**Keywords:** Superparamagnetic carbon-encapsulated iron nanoparticles, Fluorescent nanoparticles, Magnetocontrollability, Human fibrosarcoma cell line, Magnetic field, 3-Dimensional (3D) reconstruction, Electron microscopy

## Abstract

**Background:**

Theranostics application of superparamagnetic nanoparticles based on magnetite and maghemite is impeded by their toxicity. The use of additional protective shells significantly reduced the magnetic properties of the nanoparticles. Therefore, iron carbides and pure iron nanoparticles coated with multiple layers of onion-like carbon sheath seem to be optimal for biomedicine. Fluorescent markers associated with magnetic nanoparticles provide reliable means for their multimodal visualization. Here, biocompatibility of iron nanoparticles coated with graphite-like shell and labeled with Alexa 647 fluorescent marker has been investigated.

**Methods:**

Iron core nanoparticles with intact carbon shells were purified by magnetoseparation after hydrochloric acid treatment. The structure of the NPs (nanoparticles) was examined with a high resolution electron microscopy. The surface of the NPs was alkylcarboxylated and further aminated for covalent linking with Alexa Fluor 647 fluorochrome to produce modified fluorescent magnetic nanoparticles (MFMNPs). Live fluorescent imaging and correlative light-electron microscopy were used to study the NPs intracellular distribution and the effects of constant magnetic field on internalized NPs in the cell culture were analyzed. Cell viability was assayed by measuring a proliferative pool with Click-IT labeling.

**Results:**

The microstructure and magnetic properties of superparamagnetic Fe@C core–shell NPs as well as their endocytosis by living tumor cells, and behavior inside the cells in constant magnetic field (150 mT) were studied. Correlative light-electron microscopy demonstrated that NPs retained their microstructure after internalization by the living cells. Application of constant magnetic field caused orientation of internalized NPs along power lines thus demonstrating their magnetocontrollability. Carbon onion-like shells make these NPs biocompatible and enable long-term observation with confocal microscope. It was found that iron core of NPs shows no toxic effect on the cell physiology, does not inhibit the cell proliferation and also does not induce apoptosis.

**Conclusions:**

Non-toxic, biologically compatible superparamagnetic fluorescent MFMNPs can be further used for biological application such as delivery of biologically active compounds both inside the cell and inside the whole organism, magnetic separation, and magnetic resonance imaging (MRI) diagnostics.

## Background

Nanotechnology opens up new opportunities for medicine and biology and for that reason the research in this area is very dynamic [1–3]. Nanoparticles are applied for target delivery of drugs or genes, tumor cells detection, also used in diagnostic methods of different diseases, sensors, as well as markers in bioimaging and much more [4]. A particularly active area of this research field is the studies of magnetic nanoparticles (MNPs) [5–7]. They can be used for magnetic resonance imaging (MRI), hyperthermia, magnetic separation, drug delivery, etc. [8, 9]. The MNPs size, shape, composition, modifications, and magnetic properties can play an important role in the ability of nanoparticles (NPs) to interact with the cells and effect on them [10–12]. Accordingly, the current research has been focused on attempts to increase the magnetic properties of NPs for in vitro and in vivo applications. In practice, this problem is very relevant, since the responses of MNPs to the applied magnetic field in solution and inside the cells are significantly different. This is due to the fact that the cell cytoplasm has higher viscosity. Moreover, after cell penetration MNPs are localized inside endosomes/endolysosomes and later can bind with different types of cytoskeleton [13–16]. Both these facts create additional interference for MNPs movements.

The main motivation for current study has been the need for instruments for targeted delivery of MNPs-bound drugs to specific intracellular structures. The first step in this direction would be studies of fine details of intracellular routes of internalized MNPs. The approaches used in our previous works allowed us to analyze the dynamics of MNPs interaction with the cells in general, starting from their adhesion to the plasma membrane and interaction with intracellular membrane organelles to the point of their exit from the cell (endocytosis–exocytosis cycle) [17, 18]. However, some TEM observations showing localization of MNPs aggregates in the cytoplasm without any connection with membranes allowed us to suggest that the picture of the MNPs behavior in a cell is incomplete and requires additional investigation using alternative experimental approaches. Fluorescent tags seem to be convenient tools for tracing the behavior of individual MNPs, which also allows analyzing the colocalization and dynamic interactions of MNPs with specific cellular structures with high precision using superresolution optical microscopy. This is particularly important for studies involving intracellular manipulations on MNPs by external magnetic field.

The most used MNPs are iron oxide NPs, Fe_3_O_4_ (magnetite) or γ-Fe_2_O_3_ (maghemite). In our previous study we investigated iron carbide Fe_7_C_3_@C MNPs and demonstrated that they have higher value of magnetization than iron oxide-based NPs [17, 18]. However, it is obvious that NPs with pure Fe core should display even better magnetic properties making them a preferred tool for biomedical applications. This type of NPs (Fe@C) has been already tested for their surface functionalization and biomolecules binding [19–21]. However, in these works their biocompatibility and magnetocontrollability in cellular environment have not been tested. In the present study, these issues were addressed by using in vitro cellular assays.

## Results

### Fe@C nanoparticles characterization and surface modification

In our experiments we used commercial Fe@C NPs, which we purified to remove impurities (large NPs and non-coated iron NPs). Acid treatment was used for elimination of NPs with defective shells, as it was described earlier [19], along with some modification (see: “Methods”). Acid treatment of NPs surface also led to their hydrophilization [20]. Purified samples were characterized by their microstructure and size, and then the surface of NPs was modified by alkylcarboxylation and subsequent amination.

**Fig. 1:**
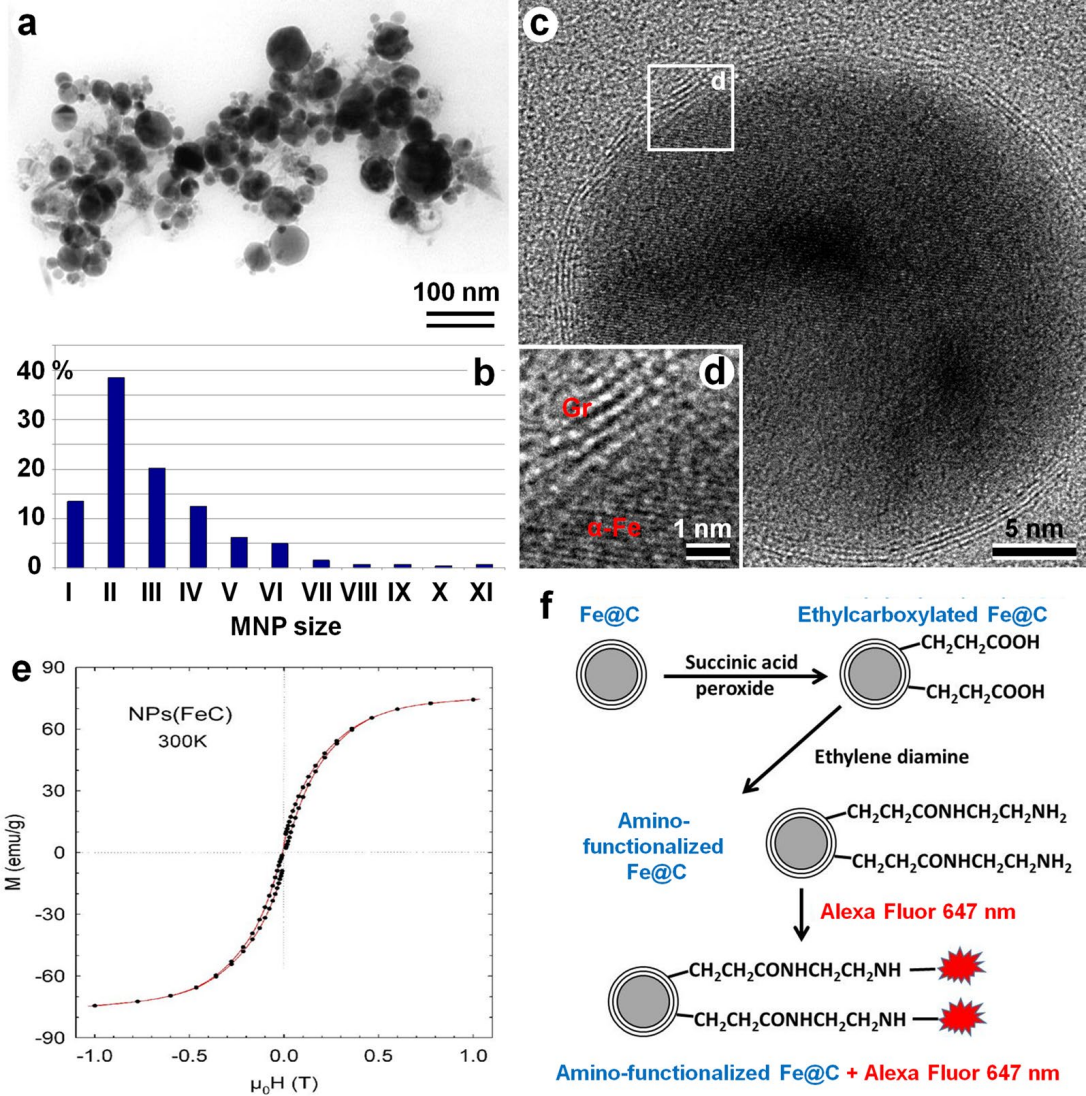
Characteristics of the initial Fe@C NPs. **a** TEM micrograph of Fe@C NPs; **b** Histogram of Fe@C NPs distribution by size. Size classes: I: < 10 nm, II: 10–20 nm; III: 20–30 nm; IV: 30–40 nm; V: 40–50 nm; VI: 50–60 nm; VII: 60–70 nm; VIII: 70–80 nm; IX: 80–90 nm; X: 90–100 nm; XI: > 100 nm; **c**, **d** HRTEM micrographs of Fe@C NPs, which demonstrate the iron lattice of graphitic-like NP shell (Gr) and alpha Fe core (α**-**Fe); **e** magnetization curves of Fe@C NPs measured at 300 K. **f** Scheme of consecutive chemical modifications of Fe@C NPs resulting in Alexa Fluor 647 binding

The initial Fe@C NPs (Aldrich, USA) were suspended in distilled water. Possible metallic impurity and all suspended nanoparticles with damaged shells were eliminated by HCl treatment. Thereafter, suspension of nanoparticles was treated by high power ultrasound to break up large aggregates and magnetically separate only *Fe@C NPs*. Transmission electron microscopy (TEM) analysis revealed that all NPs had spherical shape and consisted of an electron-dense Fe core and less electron-dense carbon shell (Fig. 1). The average size of *Fe@C NPs* was 25 nm (min 3 nm, max 363 nm; n = 505) (Fig. 1b). Microstructure of Fe@C NPs was studied by high resolution TEM (HRTEM). This analysis confirmed that *Fe@C NPs*’ core constitutes an iron lattice and the core was coated with multiple layers of onion-like carbon (Fig. 1c, d). The average size of MNPs particles studied by HRTEM was 30.3 ± 6 nm, with the core diameter being 28.1 ± 6 nm, and the shell thickness 2.2 ± 0.4 nm (N = 50). It can be noted that the shell thickness for the smallest MNPs particles (class I) was the smallest (1.3 ± 1.2 nm), and as the particle diameter increased, it gradually increased (Table 1).

**Table 1:** HRTEM analysis of core size and shell thickness of MNP

	I class < 10 nm	II class 10–20 nm	III class 20–30 nm	IV class 30–40 nm	V class 40–50 nm	VI class 50–60 nm	VII–X classes 60–100 nm
% of MNPs in purified suspension	16	28	20	12	10	8	6
Thickness of the shell, nm	1.3 ± 1.1	2.0 ± 0.2	1.9 ± 0.4	2.7 ± 0.6	2.7 ± 1.2	3.2 ± 1.2	3.7 ± 0.7

Magnetic capacity of Fe@C NPs was compared with Fe_7_C_3_@C NPs investigated in our previous study [18]. A magnetization curve (M vs. H) of Fe@C NPs obtained at 300 K showed the typical superparamagnetic behavior with a high value of magnetic saturation, 75 emu/g (Fig. 1e). This value is higher than that obtained previously for Fe_7_C_3_@C NPs (54 emu/g) [18] and for Fe@C (63 emu/g) [20]. These data thus demonstrated that commercial Fe@C NPs possess superparamagnetic properties, have high level of magnetic saturation and can be chemically modified for future biological application.

To investigate Fe@C NPs’ (even smaller 20 nm) interaction with living cells by confocal microscopy MNPs were labeled with the fluorescent dye. For this purpose the carbon shell surface of NPs was first alkylcarboxylated and subsequently aminated for covalent linking to the molecules of fluorochrome Alexa Fluor 647 (Fig. 1f).

Interactions of obtained modified fluorescent magnetic nanoparticles (MFMNPs) with living cells can be observed (even for nanoparticles smaller than 20 nm) by confocal microscopy. Moreover, we analyzed MFMNPs interaction with HT1080 human fibrosarcoma cells both in the absence of constant magnetic field and after its application by time-lapse microscopy. It was detected and confirmed by correlative confocal-transmission electron microscopy that primary MFMNPs as well as their aggregates were endocytosed by cells. Within the cells, they were able to line up along the magnetic field lines. It is important to note that MFMNPs were non-toxic for the cells: they showed no effect on cell proliferation and did not cause the cell death. Therefore, these MFMNPs, possessing higher magnetic properties than previously described iron oxide and iron carbide NPs, can be used for biological studies in vitro as well as in vivo.

### Interaction of modified Fe@C-C_5_ON_2_H_10_-Alexa Fluor 647 nanoparticles (MFMNPs) with cells

To study whether cells will interact with and absorb with endocytosis MFMNPs, we cultivated human fibrosarcoma HT1080 cells with 20 μg/ml MFMNPs during 24 h in the chamber for intravital observation. It was detected that aggregates of MFMNPs began to accumulate on the cell surface after 30 min of co-cultivation. Prolonged incubation of HT1080 culture with MFMNPs demonstrated high efficiency of MFMNPs’ internalization by cells.

**Fig. 2:**
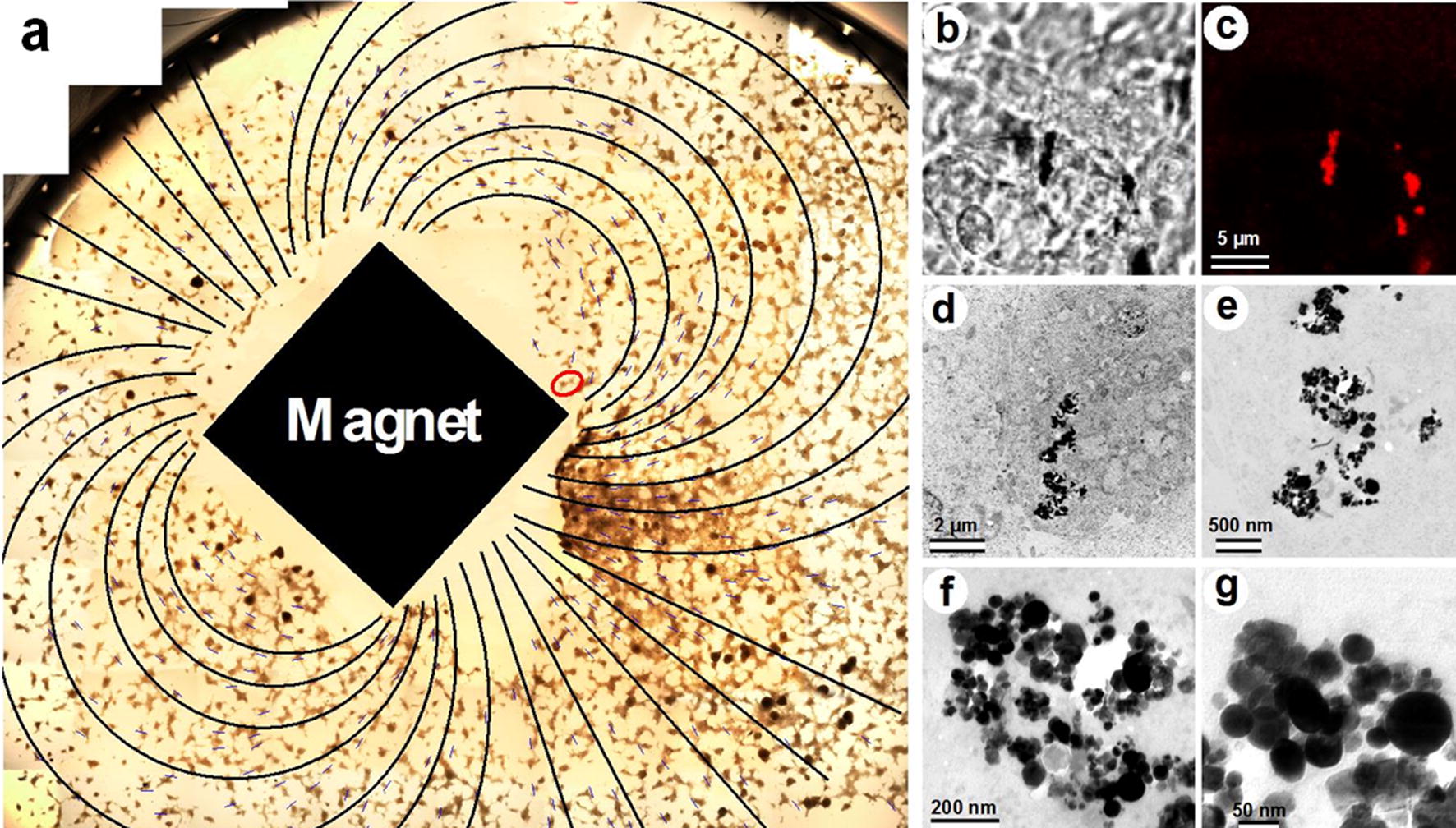
MFMNPs behavior inside the cells in a constant magnetic field exposure. **a** Light microscopy micrograph of cells incubated with MFMNPs for 24 h, then placed in magnetic field for 16 h and embedded in epoxy resin. Small blue lines repeat the arrangement of MFMNPs inside the cells in magnetic field; black lines, reconstructed on the basis of blue lines, demonstrate the magnetic field lines around the permanent magnet. **b**–**g** Correlative light-electron microscopy of the cell, marked with red oval on the panel **a**, with Alexa Fluor 647 labeled MFMNPs inside: **b** phase-contrast microscopy, **c** confocal microscopy, **d**–**g** TEM images

**Fig. 3:**
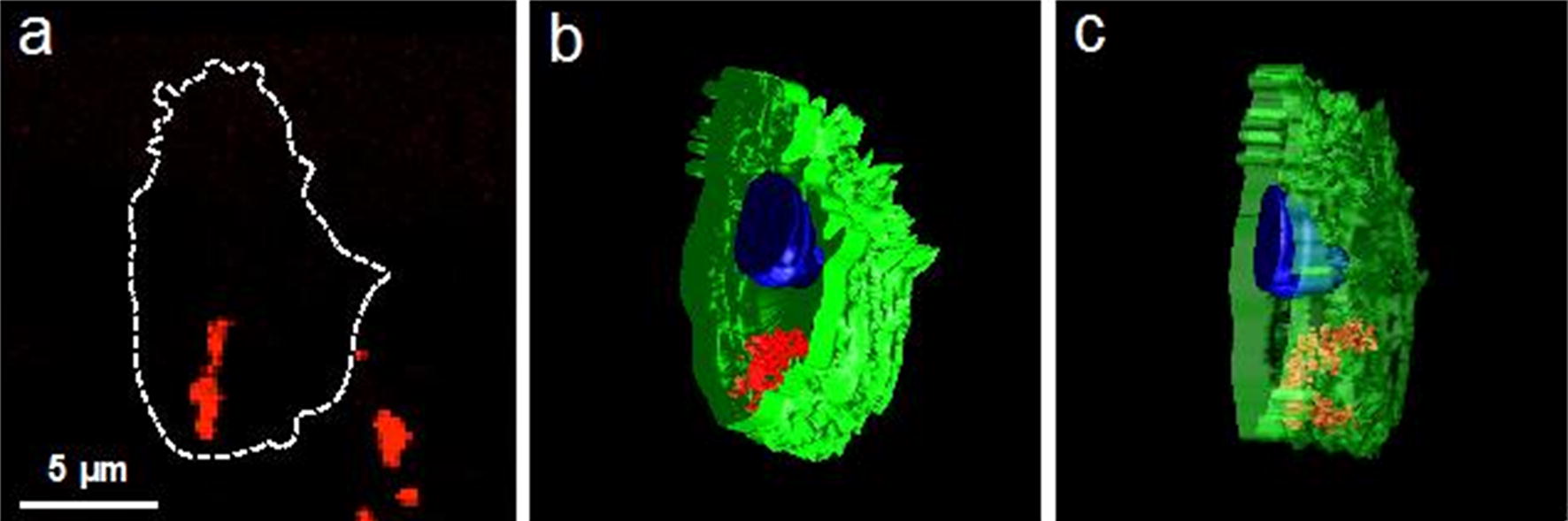
MFMNPs arrangement inside the cell in magnetic field expose. **a** Confocal micrograph of MFMNPs aggregates inside HT1080 cell after 16 h of co-cultivation in constant magnetic field exposure, the dotted line displays the cell boundaries. **b**, **c** 3D reconstructions of this cell made on the basis of serial TEM micrograph: green—cell plasma membrane, blue—cell nucleus, red—MFMNPs aggregates

The application of constant magnetic field (0,15T) to the cells after 24 h of co-cultivation with MFMNPs showed that MFMNPs’ aggregates became lined up along the magnetic field lines inside the cells (Fig. 2a). Florescence signal of Alexa Fluor 647 was precisely co-localized with MFMNPs’ aggregates visible in phase contrast microscopy (Fig. 2b, c). Intracellular localization of MFMNPs and their structure were analyzed by correlative light-electron microscopy (Fig. 2b–g). TEM study showed that aggregate of MFMNPs was localized directly in the cell cytoplasm without membrane surrounding. 3D reconstruction illustrating the MFMNPs arrangement inside the cell was prepared on the basis of TEM photos obtained from serial ultrathin sections (Fig. 3b, c). It should be noted that MFMNPs retained carbon onion-like layers on their surface, which protected the cell from pure iron influence. Therefore, no ultrastructural changes of cell organelles morphology have been detected in the cell containing core–shell iron MFMNPs after 16 h of magnetic field exposure.

Thus, our data showed that carbon-coated MFMNPs can be internalized by the cells and preserve their structure and magnetic properties inside the cell cytoplasm.

### MFMNPs do not affect the cell proliferation and death

**Fig. 4:**
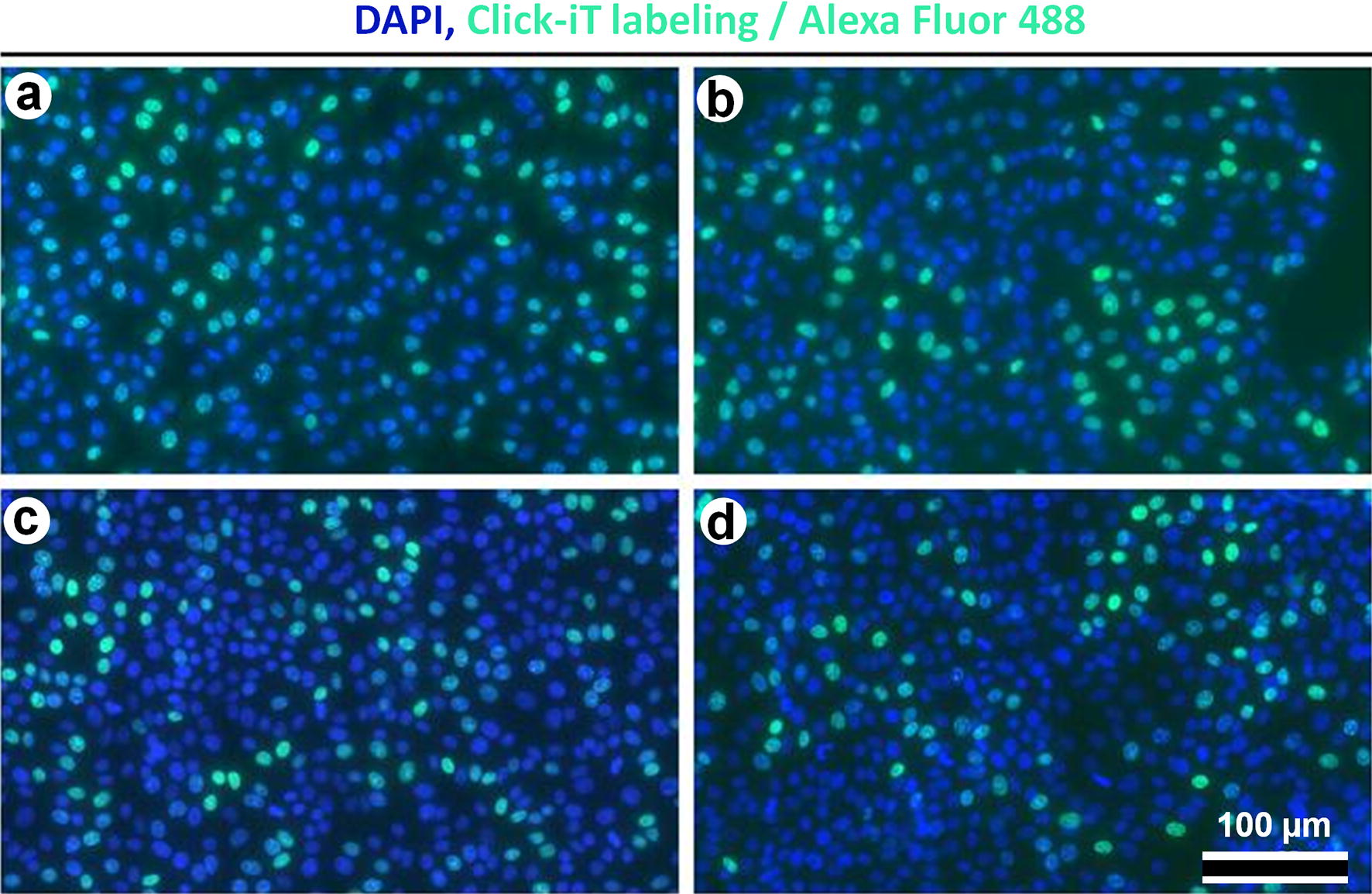
MFMNPs do not effect on cell proliferation. DAPI and Click-iT labeling of cells, fluorescent microscopy: **a** control cells; **b** cells cultivated with MFMNPs during 24 h; **c** cells incubated with MFMNPs during 48 h; **d** cells cultivated with MFMNPs during 72 h

To determine whether the core–shell MFMNPs have an adverse effect on cells we analyzed the cell proliferation after incubation with MFMNPs during 24, 48 and 72 h (Fig. 4). Method of pulsed Click-iT labeling revealed that the average percentage of cells in synthetic phase (S-phase) of cell cycle was 27.5 ± 4.1% in control culture, 26.4 ± 5% after 24 h of co-cultivation with NPs, 23.1 ± 6% after 48 h and 21.5 ± 4.6% after 72 h.

Based on the morphology of cell nuclei we also calculated the percentage of apoptotic cells in the culture. The results showed that in control cells there were 1,2 ± 0,3% of apoptotic cells, after 24 h of incubation with MFMNPs − 1.4 ± 0.3%, after 48 h − 1.9 ± 0.4% and after 72 h − 2.2 ± 0.6%.

Cell viability of HT1080 cells in presence of MFMNPs was additionally controlled by MTS assay [indirect test of mitochondrial enzyme activity by (3-(4,5-dimethylthiazol-2-yl)-5-(3-carboxymethoxyphenyl)-2-(4-sulfophenyl)-2H-tetrazolium)]. Cells were incubated for 24 h with MFMNPs (20 μg/ml) and then placed into magnetic field (detailed description in “Methods”). Viability of control cells after 40 h of cultivation was 100 ± 1.1%, cells with distilled water addition 98 ± 0.3%, cells incubated with MFMNPs 99.7 ± 1.2%, cells with MFMNPs incubated 16 h in magnetic field 98.1 ± 1.2%.

Therefore, we can conclude that the core–shell MFMNPs do not inhibit cell proliferation and do not significantly increase the level of apoptosis in culture.

### Cell ultrastructure after incubation with MFMNPs

**Fig. 5:**
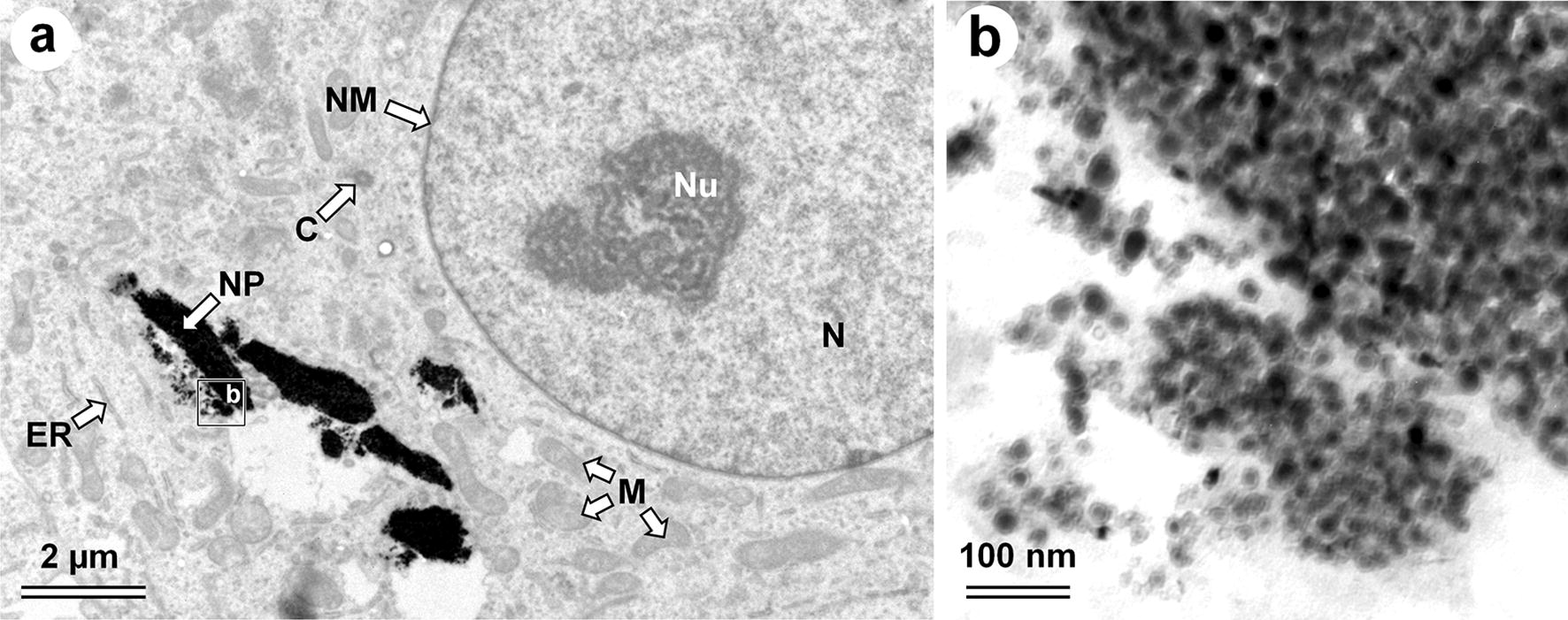
Ultrastructure of the cell after 40 h incubation with MFMNPs (last 16 h in magnetic field). **a** Small magnification TEM image. **b** High magnification of the region with MFMNPs. *C* centriole, *ER* endoplasmic reticulum, *M* mitochondria, *N* nucleus, *NM* nucleus membrane, *NU* nucleolus, *NP* aggregates of MFMNPs

Morphological analysis of cell organelles after incubation with MFMNPs was made using TEM: normal chromatin structure of the nucleus, active nucleolus with well-defined areas of granular and fibrillar components, nuclear membrane without extensions of the intermembrane space, structurally unchanged mitochondria moderately electron dense matrix (an indicator of metabolic activity) and the normal structure of the outer and inner membrane, granular endoplasmic reticulum with ribosomes (the active protein synthesis) were found (Fig. 5). So cells after 40 h incubation with MFMNPs (16 last hours in magnetic field) showed no deviations from normal morphology.

## Discussion

A number of studies reported fluorescent labeling of magnetite NPs and its application for intracellular tracking [21–23]. Carbon-shell MNPs with Fe–carbide or Fe cores have not yet been used for similar experiments, not saying about studies of their magnetocontrollability inside the cells. To pursue this goal, we created MFMNPs covalently labeled with the far-red fluorophore Alexa Fluor 647 with the aim of using them for in situ and in vivo theranostics and multimodal imaging including the confocal, superresolution and in vivo imaging approaches. Such a labeling allows (i) to visualize even very small particles using confocal microscope, and (ii) to investigate MFMNPs interaction with living cells.

The advantages of MFMNPs for these kinds of experiments seem obvious due to their small size and increased biocompatibility due to carbon onion-like shell that makes them chemically stable and virtually eliminates cytotoxicity. All these properties make MFMNPs an optimal instrument for biological applications.

The size of these MFMNPs does not exceed 100 nm that satisfies the criterion for nanomaterials [24, 25]. Moreover, the investigated MFMNPs demonstrate superparamagnetic properties and have high value of magnetic saturation. Consequently, they can allow to bypass many restrictions characteristic of superparamagnetic iron oxide nanoparticles (SPIONs), the magnetization of which is often lower [26–28], and to expand the scope of magnetic NPs application.

Multiple layers of onion-like carbon, which cover the iron cores of NPs, protect them from oxidation. The opportunity to modify their surface and label it with fluorescent molecules enables the application of these MFMNPs for in vitro as well as in vivo studies and for tracking their localization and movements in live systems.

The analysis of MFMNPs interactions with human cells revealed that they are effectively internalized by cells. Based on our previous data and results of other researchers [13, 15, 18] we can note that although the way of NPs endocytosis can vary, but in any case, the NPs become localized inside endosomes, which subsequently fuse with the lysosomes. Later, the NPs are released and localized mainly in the cell cytoplasm. TEM analysis of the MFMNPs inside the cell found in the cytoplasm after 40 h of co-incubation with the cell has demonstrated no changes in their structure. This observation means that MFMNPs are resistant to various kinds of cell effects, in particular to lysosomal enzymes. Time-lapse imaging revealed that fluorescence of MFMNPs labeled with Alexa Fluor 647 also persisted during in vitro assays.

For biological application NPs must be not toxic for cells. We demonstrated that incubation of human fibrosarcoma cells with MFMNPs during 24–72 h did not affect their cytophysiology. Decrease in the number of cells in S-phase of cell cycle after incubation with MFMNPs for 48 and more hours was insignificant compared to control cells.

In addition, the MFMNPs are capable of forming intracellular oriented aggregates aligned in the magnetic field, which points to the possibility of using these MFMNPs for directed translocation and/or retention of entire cells (for example, immune cells) to/in the centers of inflammation of tumors, for magnetoseparation and other applications that require magnetocontrollability. Previous attempts to demonstrate magnetocontrollability of magnetic Fe–carbide NPs required much stronger magnetic fields and under the experimental conditions used in this study the working range of the constant magnets was limited to only 2 mm [18], while in the present work MFMNPs were manipulated at the distance about 5 times longer.

To sum up, our results demonstrate the advantages of MFMNPs labeled with far-red fluorophores as a platform for targeted drug delivery and multimodal imaging and diagnostics due to their high level of biocompatibility and superior magnetic characteristics. These properties open broader perspectives to the use of magnetic field for long-range magnetocontrollability in biomedical application.

## Conclusions

Thus, based on our data we can conclude that MFMNPs have superparamagnetic properties and demonstrate high value of magnetic saturation that is sufficient for manipulation at the intracellular level. It is precisely these properties of such nanoparticles, combined with the possibility of chemical modification and fluorescent labeling without the appearance of cytotoxicity that allowed us to use them for the purposes indicated in this study. Moreover, these MFMNPs can be chemically modified and labeled that is useful for biological application by providing an opportunity to reliably identify MFMNPs inside the cells and in perspective inside the body of experimental animals (using intravital microscopy or IVIS). MFMNPs are endocytosed by HT1080 cells and do not affect significantly on their cytophysiology. For this reason, non-toxic, biologically compatible superparamagnetic fluorescent MFMNPs can be further used for biological application such as delivery of biologically active compounds both inside the cell and inside the whole organism, magnetic separation, MRI diagnostics.

## Methods

### Preparation of NMP fraction for biological experiments and transmission electron microscopy analysis of nanoparticles

Commercial of Fe@C NPs (Aldrich, USA) were used for this study. The portion of these NPs had either damaged or very thin carbon shells. After opening of the sealed package Fe@C NPs were immediately placed into a vial with distilled water and then treated with 1 M HCl for 24 h to remove NPs having damaged carbon shells. Thereafter, Fe@C NPs have been centrifuged (17,000*g*, 10 min) and washed 3 times by distilled water. Fe@C NPs containing iron cores have been separated from nonmagnetic fraction with the help of permanent magnet. Fe@C NPs were suspended in distilled water, treated with high power ultrasound (3 times for 2–3 min) and left settling for 1 h. MNPs from such settled suspension were centrifuged (17,000*g*, 10 min) and then transferred into a dry alcohol. 3 μl of the obtained solution were placed on the surface of Formvar and carbon coated electron microscopy grid and left to dry. The obtained sample was investigated by transmission electron microscope JEM 1011 (JEOL, Japan) equipped with a Gatan digital camera driven by Digital Micrograph software (Gatan, Pleasanton, USA) at 100 kV.

### High resolution transmission electron microscopy analysis of nanoparticles

For Fe@C NPs structure and atomic composition analysis a High resolution transmission electron microscopy) was used. Samples were prepared by crushing the powders in n-butanol and then the small crystallites in the suspension were deposited onto a holey carbon film, supported by a copper grid. The selected area electron diffraction and the high resolution electron microscopy were performed with a JEOL 2100F electron microscope operating at 200 kV.

### Nanoparticles magnetic properties measurement

300 K temperature magnetization curves of the samples were measured with a Quantum Design physical property measurement system magnetometer.

### Analysis of nanoparticles distribution by size

Based on the photograph obtained by TEM, the sizes of Fe@C NPs were measured in the ImageJ software. Scale bar was taken as standard. The percentages of Fe@C NPs with different sizes and minimum/maximum sizes of Fe@C NPs were calculated in Microsoft Excel 2007. The number of analyzed Fe@C NPs (n) was 505.

### Chemical modification of Fe@C nanoparticles surface

20 mg of Fe@C NPs black powder (Sigma Aldrich) was placed in 200 ml of dry *o*-dichlorobenzene and sonicated for 30 min. Then the mixture was heated in a N_2_ atmosphere at 110 °C for 2 days with the periodic addition of 2.5 g total of succinic acid peroxide. After reaction completion, the product was washed by pouring large quantity of chloroform and sonication for 30 min, and then filtered. The final product, succinyl functionalized Fe@C NPs, was washed on the Teflon filter multiple times with THF and ethanol. The dried product has been characterized by FTIR, TGA and EDS.

Succinyl functionalized Fe@C NPs (50 mg) was taken into 100 ml of dimethylformamide (DMF) and sonicated for about 30 min to achieve a homogenous dispersion. Ethylene diamine (1 g) was added to this solution then followed by slow addition of Dicyclohexylcarbodiimide (DCC) (3.5 g) in 10 ml of DMF dropwise over 1 h, and the resulting mixture was stirred at room temperature for 24 h. The final product was filtered through a 0.2 um polytetrafluoroethylene (PTFE) membrane and washed several time with DMF followed by chloroform and drying. The presence of terminal amino group in the final product has been established by Kaiser test and also by Fourier-transform infrared spectroscopy (FTIR). Amino-functionalized Fe@C NPs were labeled with AlexaFluor-647 Succinimidyl Ester (Thermo Fisher) according to manufacturer’s protocol (https://assets.thermofisher.com/TFS-Assets/LSG/manuals/mp00143.pdf).

### Cell culture

HT1080 human fibrosarcoma cell line (Institute of Cytology, Russian Academy of Science) was maintained in Dulbecco modified Eagle’s medium (DMEM) (Paneco) supplemented with 10% Fetal Bovine Serum (“PAA Laboratories”, Austria), 2 mM l-glutamine, and 80 μg/ml of gentamycin. Cells were seeded on glass-bottomed Petri dishes (LabTek, USA) at density of 10^5^ cells/ml and cultured at 37 °C in a humidified incubator supplied with 5% CO_2_. Experiments were conducted 48 h after cultivation.

### Live imaging

Suspension of Alexa Fluor 647-Fe@C NPs (MFMNPs) in distilled water was added to the culture medium until final concentration of 20 µg/ml. After 24 h of co-cultivation gold-plated permanent NdFeB magnet (5 × 5 × 5 mm^3^ cube, magnetic field 150 mT) was placed in Petri dish for 16 h. Cells were imaged by time-lapse confocal microscopy under 37 °C and 5% of CO_2_ for the next 24 h on an Olympus IX70 inverted microscope equipped with CCD-camera Orca-RT+ (Hamamatsu, Japan) and controlled by Micromanager 1.4 software. Illumination conditions (neutral-density filters, lamp voltage, exposure time) were set to minimize photo toxicity.

### Correlative light-electron microscopy

After vital observation the cells were washed three times with fresh serum-free pre-warmed medium to remove free MFMNPs, fixed in 2.5% glutaraldehyde in 100 mM phosphate buffer (pH 7.4) for 2 h with subsequent post-fixation in 1% OsO_4_, dehydrated in an increasing concentration of alcohols and propylene oxide and embedded in Epon (Sigma). Cells of interest, which were investigated by confocal microscope during vital observation, were found by light microscope (based on phase-contrast photos of low magnification). Serial ultrathin sections (70 nm) of these cells were prepared with Leica ultramicrotome and observed using JEM 1011 TEM. Obtained photographs were processed by Photoshop software.

Light microscopy photographs of the whole Petri dish were made and combined into a common image in Photoshop software. Lines repeating the arrangement of MFMNPs aggregates inside the cells were drawn in the Microsoft PowerPoint. On the basis of these lines, the magnetic field lines around the permanent magnet were reconstructed.

## 3D reconstruction preparing

To prepare 3D reconstruction of MFMNPs inside the cell, TEM photographs of serial sections were made and processed in Photoshop software. 3D model construction was realized by 3dmod software like it was described before [29, 30].

### Click-IT labeling of cells

5-ethynyl-2′-deoxyuridine (EdU, TermoFisher Scientific), thymidine analogue for labeling dividing cells, was added to culture medium at concentration of 1:1000 for 10 min. Then HT1080 cells were treated during 1 min in the solution of 0,1% Triton X-100, 5 mM MgCl_2_ in Phosphate-buffered saline buffer solution (PBS), and fixed with 3,7% formaldehyde solution during 15 min. After this cells were washed 3 times for 5 min with PBS (with 5 mM MgCl_2_) and incubated with 1% bovine serum albumin (BSA) during 30 min in the humid chamber. Cells were then incubated with Click-iT Assay Kit (Click-iT reaction buffer, CuSO_4_, Alexa Fluor 488 and Reaction buffer additive; TermoFisher Scientific) in the dark humid chamber during 30 min at room temperature, washed with PBS (with 5 mM MgCl_2_) 3 times for 5 min, and embedded in Mowiol with DAPI (4′,6-diamidino-2-phenylindole, Sigma). Obtained samples were investigated by Nikon C2 inverted fluorescent microscope with Andor iXon black-and-white camera, PlanApo objective (20×; aperture 0.75), and NIS-Elements AR 4.13 software.

### MTS Assay

HT1080 cells were plated at concentration of 10,000 cells per well in 96-well plate. After 48 h, culture medium in the wells was replaced with new one and MFMNPs solution in distilled water was added to the cells at final concentration 20 μg/ml (by Fe@C). Distilled water was served as negative control. Cells were cultivated for 24 h more in CO_2_-incubator. Then gold-plated permanent NdFeB magnet was placed in the neighboring empty well between the wells with cells co-cultivated with MNPs. Cells, which continued to be co-cultivated with MFMNPs without magnetic field exposure were served as control to reveal the effect of MFMNPs themselves on cells. After 16 h the growth medium was removed from each well and MTS reagent (CellTiter 96 AQueous Non-Radioactive Cell Proliferation Assay, Promega, USA) in new portion of culture medium was added to each well in the following ratio—20 μl of MTS reagent and 100 μl of culture medium. After 4 h incubation at 37 °C in darkness, 100 μl of culture medium with MTS from each well was carefully replaced in new plates to avoid the presence of MFMNPs in the analyzed solution. The absorbance of the obtained solution was measured at 490 nm using Termo Scientific Multiskan GO spectrometer. Experiments were performed in triplicates. Plotting and calculation of the standard deviation value were made using GraphPad Prism 5 software.

### Statistical analysis

The frequency of Click-iT positive and apoptotic cells was represented as a percentage (mean ± SD) of the total cells counted (n = 1000 cells). Data were obtained in three independent triplicate experiments. Plotting and calculation of the standard deviation value were made using Microsoft Excel 2007 and GraphPad Prism 5 software.

## Abbreviations

BSA: bovine serum albumin
DAPI: 4′,6-diamidino-2-phenylindole
DCC: dicyclohexylcarbodiimide
DMF: dimethylformamide
FTIR: Fourier-transform infrared spectroscopy
HT1080: human fibrosarcoma cell line
HRTEM: high resolution transmission electron microscopy
MFMNPs: Alexa Fluor 647 fluorochrome to produce Modified Fluorescent Magnetic Nanoparticles
MNPs: magnetic nanoparticles
MRI: magnetic resonance imaging
NPs: nanoparticles
PBS: phosphate-buffered saline buffer solution
PTFE: polytetrafluoroethylene
TEM: transmission electron microscopy
3D: 3-dimensional
